# Coronary CT Angiography as a Diagnostic and Prognostic Tool: Perspectives from the SCOT-HEART Trial

**DOI:** 10.1007/s11886-015-0695-4

**Published:** 2016-01-18

**Authors:** Mhairi Doris, David E. Newby

**Affiliations:** Centre for Cardiovascular Science, University of Edinburgh, 49 Little France Crescent, Edinburgh, EH16 4SB UK

**Keywords:** CT coronary angiography, Angina, Coronary heart disease, Myocardial infarction, Outcomes

## Abstract

Coronary artery disease is the leading cause of death worldwide. Many trials to date have investigated the diagnostic accuracy of coronary computed tomography angiography (CCTA) when compared to the gold standard diagnostic test, invasive coronary angiography. However, whether the use of a non-invasive anatomical test, such as CCTA, can translate into improved patient risk stratification, management and outcome has yet to be established. The Scottish COmputed Tomography of the HEART (SCOT-HEART) trial sought to address these questions and determined whether CCTA, when used in addition to standard care, could aid the diagnosis, further investigation and treatment of patients referred to the cardiology clinic with suspected angina due to coronary heart disease. In this trial, CCTA clarified the diagnosis of angina due to coronary heart disease in a quarter of patients and this led to major alterations in treatment and management that appeared to reduce the risk of subsequent coronary heart disease death or non-fatal myocardial infarction. The SCOT-Heart trial has established that CCTA is a valuable diagnostic test in patients with suspected angina pectoris due to coronary heart disease and leads to greater clarity, more focused appropriate treatments and better coronary heart disease outcomes.

## Introduction

Despite significant advances in detection and management, atherosclerotic coronary artery disease remains a leading cause of ill health and mortality throughout the world [1]. Invasive coronary angiography remains the gold standard for the diagnosis of coronary artery disease, but is an invasive investigation associated with a small risk of serious complications [2]. The adoption of an effective, safe, non-invasive strategy to diagnose and risk stratify patients in order to improve clinical outcomes is the goal of current diagnostic approaches.

The evolution of coronary computed tomography angiography (CCTA) has seen improved temporal and spatial resolution and has raised the promise of providing such an accurate non-invasive anatomical evaluation of the coronary arteries. More recently, questions have focused, not only on the diagnostic accuracy of CCTA, but also its clinical application and value in improving patient outcomes (Table 1).

**Table 1:** Summary of important CCTA studies to date

Primary aim	First author/title and year, reference number	Patients (*n*)	Population characteristics	Disease prevalence (% >50 % stenosis)	Major findings
Diagnostic accuracy of CCTA compared to invasive angiography	Budoff MJ et al. (ACCURACY) 2008 [3]	230	Typical/atypical pain. No known CAD	25	NPV of 99 % compared with invasive angiography
	Miller, J et al. (CORE-64) 2008 [4]	291	Only patients with CAC ≤600 included in primary analysis	56	90 % PPV of CCTA Lower NPV of 83 %
	Meijboom et al. 2008 [5]	360	Acute and stable anginal symptoms	68	99 % sensitivity and NPV of 97 % in detecting significant stenosis. Specificity of 64 % [5].
Triage of patients from Emergency Department (accuracy in diagnosis of ACS)	Hoffman et al. (ROMICAT) 2009 [6]	368	Acute chest pain with normal initial troponin and ECG	18.5	100 % sensitivity and NPV for diagnosis of ACS in absence of CAD [6]
	Goldstein et al. (CT-STAT) 2011 [7]	699	Low-risk acute chest pain presenting to ED	4 (>70 % stenosis) 10 (25–70 % stenosis)	54 % reduction in time to diagnosis compared with MPI, lower cost of care and no difference in adverse outcomes [7]
Prognostic Value of CCTA	Puchner et al. (ROMICATII) 2014 [8]	472	Acute chest pain. Low-risk patients	10	Presence of high-risk plaques independent predictor of ACS (OR 8.9) [8]
	Chow et al. 2010 [9]	2076	Various indications (58 % chest pain)	30	CTA measures of CAD and TPS have incremental prognostic value over clinical predictors [9]
	Hadamitzky M et al. 2013 [10]	1584	Suspected, but not known CAD	20	Severity of CAD on CTA and TPS predicted death or non-fatal MI over standard clinical risk scores during 5 years follow-up [10]
Assessing clinical effectiveness of CCTA in diagnosis, management and outcomes.	Douglas P et al. (PROMISE) 2015 [11••]	10,003	Symptomatic patients referred for investigation	11	CTA associated with fewer invasive angiograms showing no obstructive disease. Reduction in CHD death/non-fatal MI at 12 months. No difference in outcome at 25 months [11••]
	Newby DE et al. (SCOTHEART) 2015 [12••]	4146	Recent onset chest pain, suspected CHD	42	CTA clarified diagnosis of angina secondary to CHD in 1 in 4 patients [12••].

*OR* odds ratio, *MPI* myocardial perfusion imaging, *TPS* total plaque score

The Scottish COmputed Tomography of the HEART (SCOT-HEART) trial was designed as a multicenter randomised controlled trial to assess systematically the role of CCTA in the diagnosis, management and prognosis of patients referred to the cardiology clinic with recent onset stable chest pain. The main questions addressed by the SCOT-HEART trial included whether CCTA could be of value when used alongside standard care in improving not only diagnosis but also further management and patient outcomes [12••].

## CCTA as a Diagnostic Tool

### Diagnostic Accuracy

Chest pain remains an extremely frequent presentation and clinical history alone is often inadequate at accurately diagnosing and risk stratifying patients with suspected angina secondary to coronary heart disease. A report by Sekhri et al. investigated the outcome of patients referred with chest pain to cardiology clinics and found that, whilst those patients diagnosed with angina had a greater risk of cardiovascular events, patients diagnosed with non-cardiac chest pain accounted for almost one third of cardiovascular-related deaths over a median follow-up period of 2.5 years [13]. Patients who were misdiagnosed were more likely to be younger and describe atypical symptoms. This highlights the need for improved diagnostic accuracy in this group of low- to intermediate-risk patients and suggests that this population may benefit most from a detailed anatomical non-invasive investigation, such as CCTA.

The diagnostic accuracy of CCTA has been well demonstrated in previous studies [14]. Initially, such studies predominantly included high-risk patients, or small cohorts of patients in single centres, leading to concerns about the lack of evidence supporting the use of CCTA in patient groups with a lower probability of coronary heart disease and thus its generalisability in the real-world setting [15–17]. In the multicenter coronary artery evaluation using 64-row multidetector computed tomography angiography (CORE 64) study, CCTA demonstrated a negative predictive value of 83 % and positive predictive value of 91 % when used in symptomatic patients with suspected coronary heart disease and calcium scores of less than 600 [4]. In this trial population, there was a high prevalence of coronary heart disease (56 % for ≥50 % stenosis on conventional coronary angiography), implying that CCTA can provide robust diagnostic information in higher risk groups. However, the primary analyses excluded patients with calcium scores greater than 600 [4]. In a substudy of the CORE-64 trial population, the diagnostic accuracy of CCTA was also noted to be high in the evaluation of patients with suspected acute coronary syndrome as well as stable coronary heart disease [18]. Similarly, the ACCURACY study, conducted by Budoff et al. in 2008, investigated the diagnostic performance of 64-multidetector row CCTA in symptomatic patients with suspected coronary heart disease who had been referred for elective invasive coronary angiography [3]. The results demonstrated that CCTA possessed a high sensitivity for the detection of stenosis at both 50 and 70 % thresholds with a negative predictive value of 99 %. In this study, whilst the specificity of coronary heart disease obstruction detection was 83 %, the positive predictive value dropped to 48 % in the identification of severe stenosis [3].

**Fig. 1:**
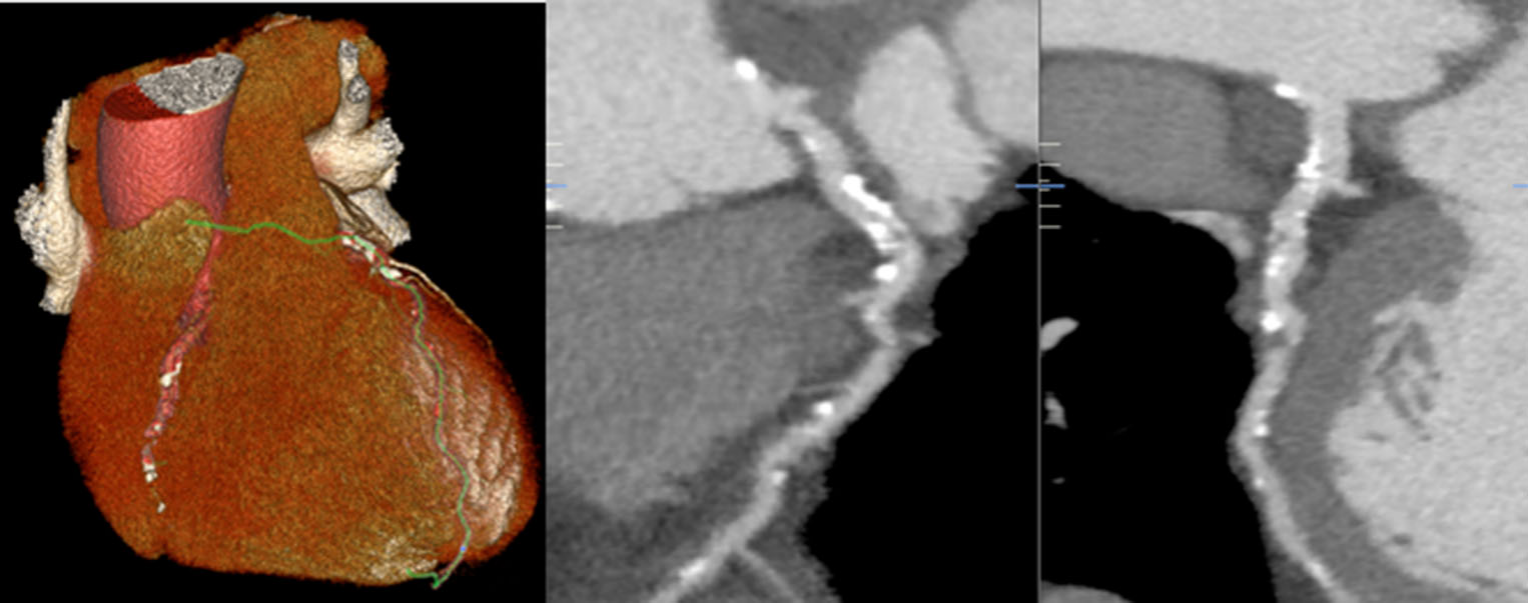
Example of coronary computed tomography angiography (CCTA) image in a 55-year-old gentleman with a calcium Agatston score of 1400

From the existing evidence, it is discernible that CCTA holds greatest value in the reliable exclusion of coronary artery disease, particularly in low- or intermediate-risk patients. Additionally, its use aids the diagnosis of coronary heart disease by reliably detecting the presence of coronary atherosclerosis. However, its reliability can falter in the accurate grading of stenosis and has been especially impeded by the over estimation of stenotic plaques, particularly in the presence of significant coronary calcification (Fig. 1). This has led to concern regarding the generalisability of CCTA as an effective diagnostic tool in the real-world population, particularly with regard to the clinical diagnosis of angina due to the suspicion of a significant incidence of false positives.

### Real-World Diagnosis of Angina

The primary objective of the SCOTHEART trial was to investigate the role of CCTA in the clinical diagnosis of angina, in order to test the generalisability of this non-invasive test in the real-world setting. The trial was intentionally designed to include a broad population of patients, truly reflective of those presenting to the cardiology clinic with stable chest pain of recent onset. Almost half of all eligible patients were recruited to the study, including patients with high calcium scores, high body mass index and atrial fibrillation. A total number of 4146 patients were randomised (1:1) to standard care alone (including cardiovascular risk assessment) or to standard care plus coronary calcium score and CCTA. Randomisation used minimisation to match for age, sex, BMI, diabetes, history of coronary heart disease, atrial fibrillation and baseline diagnosis of angina due to coronary heart disease. At baseline, cardiovascular risk was calculated using the ASSIGN score [19]. Clinicians were asked to diagnose coronary heart disease and angina secondary to coronary heart disease, as well as documenting their level of confidence in the diagnosis both at baseline and at 6 weeks follow-up. The study was powered to recruit 2069 patients per group to detect an absolute change of 4 % in the diagnosis of angina.

As opposed to comparing CCTA directly with functional testing, the SCOT-HEART trial was designed to assess the role of CCTA in addition to usual standard care, to investigate the benefit of this anatomical investigation in clarifying the diagnosis of angina secondary to coronary heart disease. In fact, the majority of patients (85 %) underwent exercise ECG prior to CCTA. The exercise ECG was abnormal in 15 % of patients [12••]. When completing the baseline assessment, the clinician diagnosed 47 % of patients as having coronary heart disease and 36 % of patients as having angina secondary to coronary heart disease. At baseline, only a small percentage of patients had a history of coronary heart disease, but there was a high prevalence of cardiovascular risk factors. Consequently, there were a large number of patients receiving preventative medications, with over 40 % of patients receiving a statin and 48 % an antiplatelet agent. The mean predicted 10-year cardiovascular risk was 17 % [12••]. Nonetheless, the majority of patients who underwent CCTA had either normal coronary arteries (38 %) or non-obstructive disease (37 %). Overall, diagnostic quality was obtained in the vast majority of patients and excellent inter-observer and intra-observer variability was documented in the diagnosis of both coronary heart disease and angina secondary to coronary heart disease [20].

For a diagnostic test to be useful, it must increase the clinician’s certainty of detecting or excluding a specific condition. At 6 weeks follow–up, the diagnosis of coronary heart disease, as defined by the attending clinician’s report, was reclassified in 27 % of patients assigned to CCTA in addition to standard care, compared with only 1 % of patients assigned to standard care alone. Similarly, the diagnosis of angina secondary to coronary heart disease changed in 23 % of patients assigned to CCTA compared to 1 % assigned to standard care. The use of CCTA doubled the certainty of diagnosing both coronary heart disease and angina secondary to coronary heart disease [12••].

In summary, the results of the SCOT-HEART trial demonstrate CCTA provides a non-invasive test that can identify or exclude coronary atherosclerosis and increase the certainty of the clinical diagnosis of angina secondary to coronary heart disease in patients presenting with recent onset stable chest pain.

### Invasive Coronary Angiography

Following previous evidence highlighting the reduced positive predictive value in assessing the severity of coronary stenosis, there is a concern that CCTA will increase the number of invasive angiograms showing non-obstructive disease. In the SCOT-HEART trial, whilst the use of CCTA was associated with a small increase in early referrals, there was no overall difference in the rates of invasive coronary angiography. In patients where CCTA triggered a new request for invasive coronary angiography, the majority (73 %) had evidence of obstructive disease and over half (58 %) underwent coronary revascularisation [12••]. Furthermore, 9 % of patients who proceeded underwent coronary artery bypass surgery, highlighting the identification prognostically significant coronary heart disease by CCTA. Indeed, long-term outcome data could help provide evidence that such alterations in downstream management driven by non-invasive imaging confer prognostic benefit.

Another large randomised trial assessing the clinical use of CCTA, the Prospective Multicenter Imaging Study for Evaluation of Chest Pain (PROMISE) study, compared the use of a diagnostic testing strategy using CCTA with functional testing (stress ECG, stress ECHO or nuclear stress testing) [11••]. The results revealed that there was a reduction in the number of invasive angiograms showing no obstructive disease in the CTA arm compared with the functional testing arm (3.4 vs 4.3 % respectively, *p* 0.02) [11••]. Overall, CCTA appeared to reduce the rates of normal invasive coronary angiography at the same time as increasing the likelihood of diagnosing important obstructive disease requiring coronary revascularisation.

### Safety

Historically, the use of CCTA has been associated with radiation doses of up to 20 mSv and this has led to concern regarding its widespread adoption as a first line investigation [21]. However, modern scanning techniques with the inclusion of radiation-lowering applications such as prospective ECG gating and the use of iterative reconstruction have markedly lowered this dose without compromising diagnostic accuracy [21–23]. In the SCOTHEART trial, the median radiation dose was 4.1 mSv, with greater than one third of this dose attributable to the measurement of the coronary calcium score [12••]. The latter did not add any additional information to that obtained by CCTA meaning that effective radiation doses are 2–3 mSv for CCTA.

In the SCOTHEART and PROMISE trials, the number of adverse reactions secondary to CCTA was very low (2 %), and all of these were recorded as mild and self-limiting [2, 11••].

As a diagnostic tool, CCTA is therefore a safe, reliable and reproducible test which can add valuable anatomical information about the coronary arterial circulation. The clinical implementation of this tool in addition to exercise electrocardiography provides a means whereby an accessible and cost-effective functional assessment can be coupled with an accurate anatomical test, clarifying the diagnosis of myocardial ischaemia secondary to coronary heart disease and reducing the need for further stress testing. By clarifying the diagnosis of angina secondary to coronary heart disease, this allows appropriate tailoring of subsequent management, including focusing invasive angiography to those in whom this is necessary. Similarly, such diagnostic clarification avoids the labelling and lifelong adherence to unnecessary medication in those patients in whom coronary heart disease is excluded. Evidence has concluded that a normal CCTA confers an excellent prognosis and extends a “warranty period” of at least 7 years [24, 25].

## CCTA as a Prognostic Tool

Following the acquisition of evidence supporting the diagnostic accuracy and clinical use of CCTA in the detection of coronary artery disease, and since the rapid development of cardiovascular imaging, there has been a drive to examine the impact of diagnostic imaging on both risk stratification and clinical outcomes.

### Risk Stratification by CCTA

Early studies examining the role of CCTA in accurate risk stratification and prognosis were largely limited to single centres and small patient cohorts [26]. In order to refine this evidence, the international multicenter CONFIRM (Coronary CT Angiography evaluation for Clinical outcomes) registry was developed and has enrolled over 30,000 patients who have undergone CCTA for evaluation of suspected coronary heart disease [26]. When examining the use of CCTA in patients without chest pain, results from the CONFIRM registry revealed that coronary artery calcium scoring and CCTA both enhanced risk stratification for all-cause mortality and a composite of all-cause mortality and non-fatal MI. However, the added value of CCTA over a model based on standard risk factors and CACS was negligible, suggesting this test could not be justified for screening of an asymptomatic population [27]. When asymptomatic patients were stratified by coronary artery calcium score, CCTA added incremental prognostic value over the Framingham Risk Score for the prediction of mortality and non-fatal MI for asymptomatic individuals with moderate coronary artery calcium scores (101–400), but not lower (<100) or higher scores (>400) [28].

In contrast, for symptomatic patients with suspected coronary heart disease, the use of CCTA measures of coronary heart disease improved discrimination of patients at risk of death or myocardial infarction when added sequentially to traditional risk models, clinical variables and coronary artery calcium score [29].

### CCTA and Prognosis

In order for a diagnostic test to alter outcomes, the results must be interpreted and clinical knowledge used to translate this information into further treatment decisions, such as the initiation and adherence to evidence-based medication. Therefore, it is challenging for a diagnostic test to have a direct effect on clinical outcomes. However, we have learned from the SCOTHEART trial that the use of CCTA leads to large changes in treatment decisions and these changes appeared to reduce the subsequent risk (hazards ratio 0.62, *p* = 0.0527) of coronary heart disease death and non-fatal MI over a median follow up period of 1.7 years. This appears to be attributable to changes in preventative therapies and coronary revascularisation [12••]. Interestingly, the results of the PROMISE trial also revealed a reduction in death and non-fatal MI in the CCTA group in a pre-specified analysis over the initial 12-month follow up period (HR 0.66, *p* = 0.049) [11••]. However, this benefit was not apparent at the completion of follow-up.

In both the SCOTHEART and PROMISE trials, the overall absolute event rates were low, reflective of the fact that the majority of patients had either normal coronary arteries or mild coronary artery disease [2, 11••]. Further research is therefore required with longer term follow-up in order to investigate more fully the effect of CCTA on clinical outcomes.

### Patient-Centred Outcomes

In the SCOTHEART trial, patients’ symptoms were reassessed at 6 weeks and there was no significant change in symptom frequency or severity between the CCTA and standard care groups. However, this was often the point at which the clinician was fully informed regarding the outcome of CCTA in this arm of the trial, and so could be considered too early to account for subsequent alterations in diagnosis or management. Longer follow-up is thus required to fully ascertain the effect of CCTA on patient-centred outcomes [12••].

### Future Perspectives

In addition to the accurate diagnosis of coronary atherosclerosis, the evolution of CCTA has enabled the non-invasive characterization of plaque morphology, which raises promise for its future in accurate risk stratification and its potential to reduce future major cardiac events. An advantage of CCTA over invasive coronary angiography is the ability to visualise the vessel wall, providing the potential to identify high-risk features of coronary plaque despite a preserved vessel lumen [30]. A meta-analysis analysing the ability of CCTA to provide quantitative measurements including vessel luminal area and coronary plaque volume showed a sensitivity of 93 % and specificity of 92 % when compared with intravascular ultrasound (IVUS) [31]. Furthermore, a meta-analysis demonstrated the ability of CCTA to differentiate low- from high-risk patients in predicting the risk of future cardiac events [32]. Indeed, Motoyama et al. demonstrated that the presence of high-risk plaques, as determined by CCTA, was an independent predictor of subsequent acute coronary syndrome [33•]. The detection of plaque progression through serial CT coronary angiography was an additional predictor of acute coronary syndrome [33•].

In addition to the detection of high-risk plaque features on CCTA, the evolution of approaches to combine this anatomical information with physiological measures of coronary blood flow and perfusion holds promise for the future of CCTA. Recent developments in the calculation of fractional flow reserve noninvasively (FFR_CT_) have been highlighted in three large multicenter studies (NXT, DISCOVER-FLOW and DeFACTO) which have compared the accuracy of FFR_CT_ with invasive FFR measurements [34–36]. A meta-analysis of the DeFACTO, NXT and DISCOVER-FLOW trials concluded that FFR_CT_ has a pooled sensitivity similar to CCTA (0.89 versus 0.89 at per-patient analysis; 0.83 versus 0.86 at per-vessel analysis) but improves specificity in both a per-vessel and per-patient analysis (0.71 versus 0.35 at per-patient analysis; 0.78 versus 0.56 at per-vessel analysis) [37]. The high negative predictive value of FFR_CT_ has raised promise for its potential to exclude ischaemia caused by intermediate grade lesions, potentially avoiding unnecessary invasive angiography [38]. Recently, the Prospective LongitudinAl Trial of FFR_CT_: Outcome and Resource Impacts (PLATFORM) study has investigated the clinical use of FFR_CT_ and the results revealed that CCTA with FFR_CT_ did in fact lead to a marked reduction in the number of invasive angiography showing no obstructive coronary artery disease [39]. This was a non-randomised study and there was no comparison with CTCA alone, and further study is therefore warranted.

Another technique which holds promise for the future potential of CT to evaluate cardiac function and physiology is the assessment of myocardial perfusion by CT. Studies comparing CT myocardial perfusion with alternative functional imaging techniques including MRI and radionucleotide perfusion imaging have demonstrated sensitivities of 83–91 % and specificities of 72–98 % for CT myocardial perfusion imaging [40]. The multicenter combined coronary angiography and myocardial perfusion by computed tomography in the identification of flow-limiting stenosis (CORE-320) study combined CCTA and myocardial CT perfusion to investigate the accuracy of CT perfusion compared to CCTA alone. The results revealed that the combination of CCTA and CT perfusion was accurate in identifying patients with flow-limiting coronary artery disease, especially in patients with no known coronary heart disease [41]. Furthermore, by stratifying the CORE-320 population according to pre-test probability of coronary artery disease, the results demonstrated that the use of combined CT perfusion and CCTA added incremental diagnostic accuracy amongst patients with high calcium scores [42]. Nonetheless, important limitations for CT perfusion imaging remain, including the impact of motion artefact and beam hardening on image interpretation as well as concerns regarding radiation dose [40]. With its evolution, this technique has the potential to develop as a valuable and accessible clinical tool alongside CCTA.

## Conclusions

CCTA continues to evolve as a rapidly developing imaging modality and has the ability to clarify the diagnosis of angina secondary to coronary heart disease. Results from the SCOTHEART trial have highlighted that routine application of CCTA, in addition to other clinical tools including exercise ECG, helps guide patient management, select appropriate treatments and appears to improve clinical outcomes including myocardial infarction. The potential ability of CCTA to evaluate and provide in depth information about plaque morphology could lead to the identification of specific high-risk characteristics within the vulnerable plaque, allowing sophisticated and accurate risk stratification. Furthermore, the evolution of these methods to combine physiological evaluation with CCTA raise the promise for the adoption of a single imaging platform to provide accurate diagnostic information, guide management and ultimately improve cardiovascular outcomes.
